# Self‐control in crows, parrots and nonhuman primates

**DOI:** 10.1002/wcs.1504

**Published:** 2019-05-20

**Authors:** Rachael Miller, Markus Boeckle, Sarah A. Jelbert, Anna Frohnwieser, Claudia A. F. Wascher, Nicola S. Clayton

**Affiliations:** ^1^ Department of Psychology University of Cambridge Cambridge UK; ^2^ Department of Cognitive Biology University of Vienna Vienna Austria; ^3^ Department of Psychotherapy Bertha von Suttner Private University Austria; ^4^ School of Life Sciences Anglia Ruskin University Cambridge UK

**Keywords:** comparative cognition, corvids, delayed gratification, parrots, self‐control

## Abstract

Self‐control is critical for both humans and nonhuman animals because it underlies complex cognitive abilities, such as decision‐making and future planning, enabling goal‐directed behavior. For instance, it is positively associated with social competence and life success measures in humans. We present the first review of delay of gratification as a measure of self‐control in nonhuman primates, corvids (crow family) and psittacines (parrot order): disparate groups that show comparable advanced cognitive abilities and similar socio‐ecological factors. We compare delay of gratification performance and identify key issues and outstanding areas for future research, including finding the best measures and drivers of delayed gratification. Our review therefore contributes to our understanding of both delayed gratification as a measure of self‐control and of complex cognition in animals.

This article is categorized under:

Cognitive Biology > Evolutionary Roots of Cognition
Psychology > Comparative Psychology

## Graphical abstract

Examples of delayed gratification tasks. (a) Exchange task with a corvid: subject can choose to swap a token (e.g., bottle top) for a reward after a delay; (b) Intertemporal choice: monkey can select the immediately available reward or wait for the delayed reward from a rotating tray.

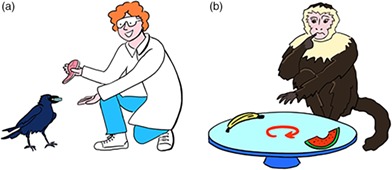

## 1 INTRODUCTION

We provide a comprehensive overview of *self‐control* (see Box 1 for a Glossary of terms, which are highlighted in the text in italics) in animals, focussing specifically on *delay of gratification* ability as a measure of self‐control. We discuss methodological issues, including whether it is possible to make species comparisons as the field currently stands. We suggest future research directions, including how best to test delayed gratification in different species, and consequently to examine the potential evolutionary drivers of delayed gratification.

BOX 1 **GLOSSARY OF DELAY OF GRATIFICATION TASKS AND RELATED TERMINOLOGY**

*Accumulation task*:

Food rewards accumulate within reach until the subject chooses to consume them.

*Exchange task*:

Subjects keep a small food reward (quantity) or a less preferred food reward (quality) until a time delay has passed. After the time delay, the food reward can be exchanged with the experimenter to obtain more of the same type of food (quantity) or a more preferred food item (quality).

*Hybrid delay task*:

Subjects choose between an immediate smaller reward and a larger but temporally delayed reward. If they choose the larger delayed reward, the experimenter starts to accumulate the rewards at a fixed rate, stopping when the subject eats the food.

*Inhibitory control*:

The ability to control reflexive, conditioned or otherwise learned responses in order to choose a conflicting, more rewarding or complex course of action (behavioral inhibition) or course of cognition (cognitive inhibition).

*Intertemporal choice task*:

Participants choose between a shorter, immediate reward in comparison to a larger, delayed one.

*Motor‐self regulation*:

Is synonymous to behavioral inhibition (see Inhibitory Control).

*Patch‐leaving task*:

Participants choose to stay or leave a patch, whereby staying results in a shorter delay and a reward that reduces in quality or quantity in subsequent staying choices; leaving the patch results in a longer delay and no reward but resets the reward to its maximum quantity or quality.

*Self‐control*:

The capacity to suppress immediate drives in favor of delayed rewards.

We have chosen three distantly related taxa (corvids, psittacines, and nonhuman primates), because they generally perform well in a suite of complex cognitive tasks (Emery & Clayton, 2004; Lambert, Jacobs, Osvath, & von Bayern, 2018). Similar delayed gratification abilities in these groups would indicate that this cognitive ability has evolved repeatedly under similar socio‐ecological pressures despite considerable evolutionary divergence in their ancestry (Seed, Emery, & Clayton, 2009; van Horik & Emery, 2011). These comparisons will also contribute to the understanding of human cognition and delayed gratification ability in humans specifically, through providing a better appreciation of the underlying factors influencing individual differences in cognitive abilities (Völter, Tinklenberg, Call, & Seed, 2018). Furthermore, by eliminating the influence of human morality, social norms and language that may contribute to choice behavior, it is possible to ascertain which cognitive and emotional processes contribute to decision‐making when faced with tempting, but ultimately short‐sighted, outcomes (Beran, 2013). To date, we are not aware of any reviews on delayed gratification that compare corvids, psittacines, and nonhuman primates as distantly related groups (though see a recent more general review of self‐control (Beran, 2018)).

### 1.1 What is delayed gratification and why is it important?

Controlling impulsive behavior is an essential contributor to culturally suitable conduct in humans, enabling successful function of our societies (Steelandt, Thierry, Broihanne, & Dufour, 2012). *Inhibitory control* is the ability to inhibit a strong, prepotent response that is likely to be tempting to execute but counterproductive to achieving a future goal (Duque & Stevens, 2017; Kabadayi, Taylor, von Bayern, & Osvath, 2016). It is an integral part of executive functioning, along with working memory and cognitive flexibility (Diamond, 2013). It includes *motor self‐regulation*—stopping a prepotent but counter‐productive movement—which underpins self‐control (Beran, 2015). Self‐control is the ability to withhold an immediate response towards a present stimulus in favor of a later stimulus, and entails the control of behavior, emotions and cognition (Nigg, 2017). This includes delay of gratification, which involves obtaining a more valuable outcome in the future, through tolerating a delay or investing a greater effort (or both) in the present (Beran, Rossettie, & Parrish, 2016).

Delay of gratification is cognitively challenging, as humans and nonhuman animals temporally discount or devalue future rewards (Loewenstein, Read, & Baumeister, 2003). Temporal discounting is usually considered to be adaptive, as several risks and costs are associated with a delay of gratification. These may include: 1. opportunity costs, for example, while waiting, individuals lose time that could be used for other activities, 2. collection risk, for example, the anticipated reward may become unavailable before it can be collected, 3. interruption risk, for example, another animal might get to the food first, 4. termination risk, for example, the chance to get the reward will be cut short, perhaps by a predator (Fawcett, McNamara, & Houston, 2012). Several theoretical models predict the rate of temporal discounting, for example, exponential discounting would predict a delayed reward to be discounted at a constant rate, whereas hyperbolic discounting predicts the discounting rate to decrease over time, with high discounting at short delays and a lower rate at longer delays. Empirical evidence suggests animals do not discount future rewards exponentially but rather, for example, at a hyperbolic rate (Ainslie, 1974; Green & Myerson, 1996; Mazur, 1988).

Self‐control underlies effective decision making, future planning, and the achievement of goal‐directed behavior (Diamond, 2013; McCormack & Atance, 2011; Santos & Rosati, 2015). Motor‐self regulation is thought to be less cognitively demanding than delay of gratification (Nigg, 2017). We note that one limiting issue in the literature at present is the variable use of terminology. For example, some authors use “self‐control” as an umbrella term that includes delayed gratification and motor self‐regulation tasks (MacLean et al., 2014). While others—particularly in the human literature (Nigg, 2017)—would consider these terms separately for the reasons described in this review, and would therefore consider delayed gratification, though not motor‐self regulation, to be a measure of self‐control.

In children, delayed gratification ability has typically been tested using the classic “marshmallow” tests, where subjects are required to choose between a small reward available immediately and a larger reward available later (Mischel, Shoda, & Rodriguez, 1989). Delayed gratification ability is influenced by socio‐ecological factors in children. For instance, children that showed better self‐control were likely to be healthier and wealthier than those with poorer self‐control (Moffitt et al., 2011). Furthermore, the stability of the environment can influence waiting times in delayed gratification tasks, with shorter waiting times in unreliable environments compared with reliable ones (Kidd, Palmeri, & Aslin, 2013).

In humans, delayed gratification performance shows high individual variation, correlates with behavioral problems (Petry, 2001), like substance abuse and gambling (Madden, Petry, Badger, & Bickel, 1997), and with measures of success in later life, such as social and academic competence, for example, (Mischel et al., 1989). However, note that a recent study called into question the reliability of the marshmallow task as a predictive measure of self‐control for adult behavior. Specifically, the study, with a more representative sample across US society than earlier studies, found no correlation between delay of gratification performance in this task as a child and subsequent behavior as a teenager once family and parental education were accounted for (Watts, Duncan, & Quan, 2018).

Self‐control is also important for nonhuman species in a variety of social and foraging contexts. For example, it is seen as an important cognitive prerequisite of cooperation, when individuals help a conspecific now in order to receive a favor in the future (Stevens & Hauser, 2004). Also, when in the presence of a stronger or more dominant competitor, an animal may inhibit their behavior by avoiding an immediate, direct approach to food or a potential mate (Duque & Stevens, 2017). For instance, one female chimpanzee (*Pan troglodytes*) learned to inhibit going to a hidden food that only she knew about when a dominant male could take that food (Menzel, 1974). In species that cache, that is, hide food for later, individuals must inhibit eating food items immediately. Some species utilize strategies requiring self‐control to prevent others from stealing their caches, including waiting to make or move a cache until the competitor is out of sight (Bugnyar & Kotrschal, 2002; Emery & Clayton, 2001). In humans and chimpanzees, general intelligence is linked with self‐control (Beran & Hopkins, 2018; Mischel et al., 1989). As in humans, overcoming impulsive behavior can be difficult for other animals (Shifferman, 2009), and may be influenced by socio‐ecological factors, like sociality (Bond, Kamil, & Balda, 2007).

All three components of executive function—working memory, cognitive flexibility and inhibitory control—have been investigated comprehensively in humans (Diamond, 2013; Nigg, 2017), and were recently advocated as a suitable starting point for comparative psychometrics (Völter et al., 2018). Here, we focus on delayed gratification as a measure of self‐control. Given that it underlies other cognitive abilities, understanding self‐control is important when interpreting cross‐species comparisons of cognitive performance (Box S1). We focus on delayed gratification, rather than motor‐self regulation because motor‐self regulation presents more “reflex‐like” aspects, like inhibiting an action, which does not necessarily require a conscious decision, though is relevant from a practical perspective for solving cognitive tasks. By contrast, delayed gratification requires an active decision to choose one of two options, hence is particularly interesting from a comparative cognition perspective, as a means to investigate the evolution of self‐control in respect to social‐ecological factors.

### 1.2 Corvids, psittacines, and nonhuman primates

**Figure 1:**
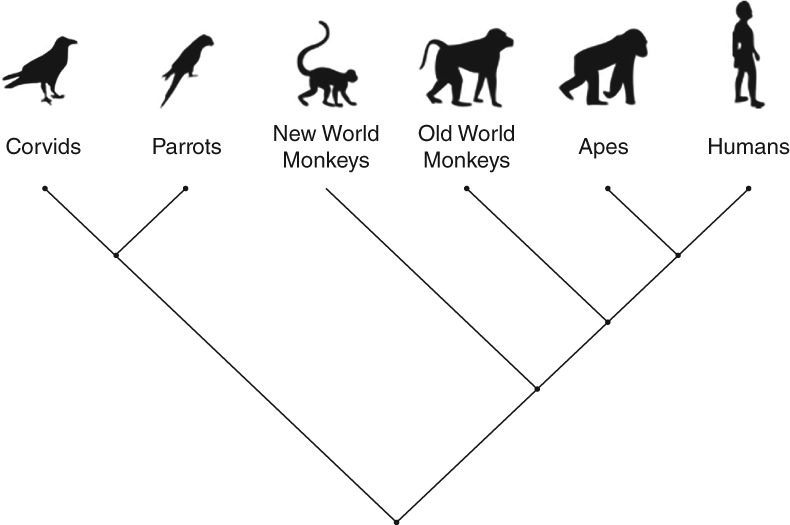
Schematic representation of a phylogenetic tree showing taxa included within this review, in order to show relative distance of relatedness. Roughly 296 million years ago (mya), birds and mammals diverged. Parrots and corvids diverged roughly 96.4 mya. New world monkeys diverged 35 mya, old world monkeys 25 mya, and great apes between 12 and 6 mya from human evolution

Corvids (crow family) and psittacines (parrot order) shared a common ancestor with primates over 300 million years ago (Figure 1). Despite this, these disparate groups often show comparable performance in many cognitive tasks (Emery & Clayton, 2004; Lambert et al., 2018), which is an example of convergent evolution, where external socio‐ecological pressures lead to similar traits developing independently in distantly related species (Emery & Clayton, 2004; van Horik, Clayton, & Emery, 2012). Different corvid and psittacine species have been shown to plan for the future, for example, Eurasian jays (*Garrulus glandarius*), California scrub‐jays (*Aphelocoma californica*) (Cheke & Clayton, 2011; Raby, Alexis, Dickinson, & Clayton, 2007), form abstract concepts, for example, African gray parrots (*Psittacus erithacus*) (Pepperberg, 2013), manufacture and/or use tools, for example, New Caledonian crows (*Corvus moneduloides*), rooks (*Corvus frugilegus*), Goffin's cockatoos (*Cacatua goffiniana*), Greater vasa parrots (*Coracopsis vasa*), Eurasian jays (Auersperg, Von Bayern, Gajdon, Huber, & Kacelnik, 2011; Bird & Emery, 2009; Cheke, Bird, & Clayton, 2011; Goodman, Hayward, & Hunt, 2018; Hunt, 1996; Lambert, Seed, & Slocombe, 2015; Rutz et al., 2016), solve complex problems through causal reasoning, for example, common ravens (*Corvus corax*), New Caledonian crows (Schloegl, Schmidt, Boeckle, Weiß, & Kotrschal, 2012; Taylor, Hunt, Medina, & Gray, 2009), keep track of who is watching when they cache their food, for example, California scrub‐jays (Dally, Emery, & Clayton, 2006; Emery & Clayton, 2001) and know when to conceal auditory information, for example, Eurasian jays, California scrub‐jays (Shaw & Clayton, 2013, 2014; Stulp, Emery, Verhulst, & Clayton, 2009).

Like many primate species, corvids and psittacines species vary in sociality (Amici, Aureli, & Call, 2008; Clayton & Emery, 2007; Lambert et al., 2018; Sussman, Garber, & Cheverud, 2005). They vary from pairs—such as Eurasian jays—to large flocks—such as rooks and jackdaws (*Coloeus monedula*)—often displaying high fission‐fusion dynamics, for example, common ravens, carrion crows (*Corvus corone*), monk parakeets (*Myiopsitta monachus*) (Hobson, Avery, & Wright, 2014; Loretto et al., 2017; Uhl et al., 2018). This requires them to interact and co‐operate with conspecifics, for example, New Caledonian crows, kea (*Nestor notabilis*), rooks, African gray parrots (Heaney, Gray, & Taylor, 2017; Péron, Rat‐Fischer, Lalot, Nagle, & Bovet, 2011; Schwing, Jocteur, Wein, Noë, & Massen, 2016; Seed, Clayton, & Emery, 2008), to remember others' identities, for example, ravens (Boeckle & Bugnyar, 2012), and even manipulate others' behavior, for example, ravens, Eurasian jays, California scrub‐jays (Bugnyar & Kotrschal, 2002; Emery & Clayton, 2004; Shaw & Clayton, 2013, 2014; Stulp et al., 2009). In some species, social systems are influenced by age and season, for example, ravens (Loretto et al., 2017).

Corvids and psittacines have some of the largest relative brain sizes of all birds (Emery & Clayton, 2005). Many of the socio‐ecological pressures implicated in primate brain evolution could be equally applicable to corvids (Seed et al., 2009) and psittacines (Lambert et al., 2018). Species within these groups are typically omnivorous foragers and exploit a range of different food sources, with some engaging in extractive foraging. They face food availability variation, and most corvid species engage in some degree of caching to overcome periods of food scarcity. Like primates, some species manufacture and use tools in the wild, such as New Caledonian crows (Hunt, 1996), while others' demonstrate tool use in captivity, such as Goffin's cockatoos (Auersperg, Szabo, von Bayern, & Kacelnik, 2012).

Arguably, some of the apparently sophisticated cognitive abilities indicated in these three groups require good self‐control, as this ability likely underlies their demonstrated capacities for decision‐making and future planning. However, while self‐control, including delayed gratification, has been investigated extensively in primates, there are few studies in corvids and psittacines, on a very limited number of species: common ravens, carrion crows, blue jays (*Cyanocitta cristata*), California scrub‐jays, pinyon jays (*Gymnorhinus cyanocephalus*), African gray parrots, kea, and Goffin's cockatoos. These species show similar socio‐ecology, for instance, most are group‐living, hence restricting evaluation of the influence of these factors on self‐control. There is a particular lack of comparative studies between primates and birds, despite their similarities in performance in other cognitive tasks and in natural history. Comparing species from distant taxa furthers our understanding of the evolution of species differences in self‐control, based on possible similarities and differences in socio‐ecological factors. It also allows us to examine the evolution of cognition through convergence (van Horik et al., 2012) and the role of proximate mechanisms underlying cognition, like inhibitory control (Osvath, Kabadayi, & Jacobs, 2014). Here, we argue that these groups provide a unique opportunity to investigate specific socio‐ecological factors shaping the evolution for self‐control. However, before this can be tested, it is necessary to review the field as it currently stands, and consider any issues preventing valid species comparisons, which we aim to do here.

## 2 DELAY OF GRATIFICATION TASKS AND PERFORMANCE

Various tasks have been used to investigate delayed gratification in nonhuman animals. These tasks fall under two broad categories: 1. *delay choice* tasks, whereby subjects are required to make dichotomous choices between immediate and delayed reward options; 2. *delay maintenance* tasks, whereby subjects need to sustain the decision to delay gratification when the immediate reward is present or already possessed during the delay (Ainslie, 1974; Beran & Evans, 2006, 2006; Evans & Beran, 2007; Tobin, Chelonis, & Logue, 1993; Tobin, Logue, Chelonis, Ackerman, & May, 1996). The key difference between these tasks is that the latter offers opportunities to study mechanisms mediating delay periods, like individual strategies to cope with delay. Delayed gratification tasks typically use rewards differing in quality (trading one food for another more preferred food) or quantity (trading a small piece of food for more of the same food) in relation to a temporal dimension. As far as the existing literature allows, we present an overview of delayed gratification tested in corvids, psittacines and nonhuman primates (Table 1), focussing primarily on the *exchange, accumulation* and *intertemporal choice* tasks. We also describe associated distraction techniques (Box S2), and exchange performance in a social context (Table S1).

**Table 1:** Overview of delayed gratification tasks tested in corvids, psittacines and nonhuman primates

Species	Exchange	Accumulation	Intertemporal choice	Hybrid delay	Patch‐leaving	References
*Corvid*						
Common raven *Corvus corax*	X	X				(Dufour, Wascher, Braun, Miller, & Bugnyar, 2012; Hillemann, Bugnyar, Kotrschal, & Wascher, 2014)
Carrion crow *Corvus corone*	X	X				(Dufour et al., 2012; Hillemann et al., 2014)
California scrub‐jay *Aphelocoma californica*			X		X	(Clayton, Dally, Gilbert, & Dickinson, 2005; Thom & Clayton, 2014)
Blue jay *Cyanocitta cristata*					X	(Stephens & Dunlap, 2009)
Pinyon jay *Gymnorhinus cyanocephalus*					X	(Stephens & Anderson, 2001; Stevens, Kennedy, Morales, & Burks, 2016)
*Psittacine*						
Goffin's cockatoo *Cacatua goffiniana*	X					(Auersperg, Laumer, & Bugnyar, 2013)
Kea *Nestor notabilis*	X					(Schwing, Weber, & Bugnyar, 2017)
African gray parrot *Psittacus erithacus*	X	X				(Koepke, Gray, & Pepperberg, 2015; Vick, Bovet, & Anderson, 2010)
*Nonhuman primate*						
Chimpanzee *Pan troglodytes*	X	X	X	X		(Amici et al., 2008; Beran & Evans, 2006, 2006; Beran & Hopkins, 2018; Beran, Savage‐Rumbaugh, Pate, & Rumbaugh, 1999; Dufour, Pelé, Sterck, & Thierry, 2007; Evans, Beran, Paglieri, & Addessi, 2012; Osvath & Osvath, 2008)
Orangutan *Pongo abelii*		X	X			(Amici et al., 2008; Osvath & Osvath, 2008; Parrish et al., 2014)
Bonobo *Pan panicus*			X			(Amici et al., 2008; Rosati, Stevens, Hare, & Hauser, 2007)
Gorilla *Gorilla gorilla*			X			(Amici et al., 2008; Stevens, 2014)
Brown capuchin *Sapajus apella*	X	X	X	X		(Amici et al., 2008; Anderson, Kuroshima, & Fujita, 2010; Bramlett, Perdue, Evans, & Beran, 2012; Evans et al., 2012; Paglieri et al., 2013; Pelé, Micheletta, Uhlrich, Thierry, & Dufour, 2011)
Tonkean macaque *Macaca tonkeana*	X	X				(Pelé et al., 2011)
Long‐tailed macaque *Macaca fascicularis*	X	X				(Amici et al., 2008; Pelé, Dufour, Micheletta, & Thierry, 2010)
Rhesus macaque *Macaca mulatta*		X				(Evans & Beran, 2007)
Squirrel monkey *Saimiri sciureus*		X				(Anderson et al., 2010)
Common marmoset *Callithrix jacchus*			X			(Rosati, Stevens, & Hauser, 2006; Stevens, Hallinan, & Hauser, 2005)
Cotton‐top tamarin *Saguinus oedipus*			X			(Rosati et al., 2006; Stevens, Hallinan, et al., 2005)
Spider monkey *Ateles geoffroyi*			X			(Amici et al., 2008; Stevens, 2014)
Black & white ruffed lemur *Varecia variegata*			X			(Stevens & Mühlhoff, 2012)
Red‐ruffed lemur *Varecia rubra*			X			(Stevens & Mühlhoff, 2012)
Black lemur *Eulemur macaco*			X			(Stevens & Mühlhoff, 2012)

*Note*. Species studied with each task denoted with “X”. This table encompasses all corvid and psittacine studies available identified through a systematic review. The primate list is not exhaustive, with studies selected to provide relevant examples for potential comparison across groups.

### 2.1 Delay maintenance: exchange and accumulation tasks

In exchange tasks, subjects need to inhibit selecting an immediate reward for a delayed one (Beran et al., 1999; Ramseyer, Pelé, Dufour, Chauvin, & Thierry, 2006). They can swap an initial, immediately available reward (e.g., 1 piece of food) for a predetermined later, preferred reward (e.g., 2 pieces of food). To test delayed gratification, the delay before receiving the preferred reward will increase incrementally. During the waiting period, the delayed reward is usually visible, and the subjects are therefore aware of what they are waiting for. The waiting period and end of this period may be indicated to the subject (e.g., by an experimenter's gesture). The outcome measure is typically the waiting time duration that the subject can refrain from selecting or eating the initial reward, and successful trial frequency/percentage. A trial ends with a subject exchanging the initial item at the end of the waiting period or consuming the initial item at any time during the trial.

In accumulation tasks, food items accumulate at a steady rate within subject reach until they touch or begin to consume them (Beran, Perdue, et al., 2016). Thus, there is a trade‐off between how long the animal waits to start eating, and the total amount of reward. In most studies, the food that an individual could gain per trial is visible in an out‐of‐reach container or shown to the subject ahead of each trial. There are two slightly different versions of this task. 1. Food items accumulate at a fixed delay (e.g., every 2 s), typically accumulating to large numbers (30/50 items), for example, in primate (Addessi et al., 2013; Beran, 2002; Evans & Beran, 2007; Pelé et al., 2011) and gray parrot (Vick et al., 2010) studies. The outcome measure is usually the number of items accumulated before the subject takes the reward. Individuals may be able to gain many rewards (e.g., (Addessi et al., 2013)). 2. Food items are transferred at an incrementally increasing delay into subject reach (e.g., corvids: (Hillemann et al., 2014), primates (Anderson et al., 2010)). The outcome measure is usually the maximum delay that individuals can endure.

### 2.2 Delay choice: inter‐temporal choice

Intertemporal choice tasks are similar to the exchange task though without the exchange component and typically require close interaction with experimenter, as subjects can wait for a better reward, or press a button to receive an immediate, less preferred reward. These tasks have been broadly applied across species, for example, via two‐key operant tasks to investigate the phenomenon of temporal discounting, that is, the devaluation of rewards over time, including in primates (Addessi et al., 2014; Rosati et al., 2007; Stevens & Mühlhoff, 2012), domestic fowl (*Gallus gallus domesticus*) (Abeyesinghe, Nicol, Hartnell, & Wathes, 2005), pigeons (*Columba livia domestica*) (Grosch & Neuringer, 1981; Logue, Chavarro, Rachlin, & Reeder, 1988) and rats (*Rattus norvegicus domesticus*) (Reynolds, De Wit, & Richards, 2002). The outcome measure is typically at which point of delay subjects equally value the immediate and delayed reward (Stevens, Hallinan, et al., 2005). The “discounting rate”, describing the steepness of the discounting function, varies considerably between species and contexts (Green, Myerson, Holt, Slevin, & Estle, 2004; Green, Myerson, & McFadden, 1997; Mazur, 1987). In humans, it correlates with cooperation frequency, with individuals who strongly devaluate future rewards cooperating less (Harris & Madden, 2002). For a critical overview of temporal discounting using intertemporal choice tasks in animals, see a recent review by Hayden (2016).

### 2.3 Strengths and weaknesses of delayed gratification tasks

A major advantage of delay maintenance tasks over delay choice tasks is that they enable investigation of the mechanisms mediating delay periods, such as individual coping strategies. A main difference between the exchange task and accumulation task is that in the exchange task, the subject has the initial reward for the entire delay period, which adds a perceived loss or endowment effect (Lakshminaryanan, Chen, & Santos, 2008) and may make this task more challenging. Disadvantages of the delay maintenance tasks are the substantial amount of training required (particularly for the exchange task) and typically close interactions with a human experimenter, making the paradigms potentially biased towards individuals/species well habituated to human presence. Indeed, familiarity with the experimenter has been found to influence performance in crows and ravens in other cognitive tests (Cibulski, Wascher, Weiß, & Kotrschal, 2014). Whereas, a main advantage of delay choice tasks is that they do not require extensive training and can be applied via operant tasks in wild and less habituated animals.

Further issues to consider are the ecological relevance of each task, as recent theoretical ideas have suggested that an animal's intertemporal preference may vary according to this (Stephens & Dunlap, 2009). For instance, the exchange task might be critiqued for whether it emulates a “real‐life” problem. However, equally, more “artificial” tasks may provide a novel test of animal flexibility or their ability to make adaptive choices in novel contexts. We outline several alternative paradigms for testing delayed gratification that present both more “natural” and artificial task variants in the other delayed gratification tasks for future comparisons section. These tasks have typically been tested in fewer species so far, or only in birds *or* primates, though not yet comparatively.

### 2.4 Task performance

**Figure 2:**
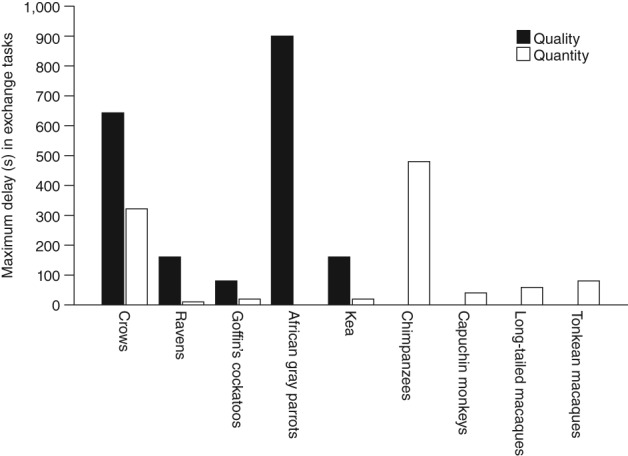
Maximum tolerated length of delays (by any individual) in exchange tasks when rewards differ in either quality (black) or quantity (white) (references in Table 1). Note that, unlike the other studies, in the African gray parrot study, the subject had been trained to respond to a “wait” command and was verbally instructed to wait in the test—other gray parrots were unable to wait more than a few seconds (Vick et al., 2010)

We present an overview of exchange and accumulation performance (Figure 2; Table 2; Table S2) and intertemporal choice performance (Table S3). As only one corvid species has been tested on the latter delay choice task, we focus on species comparisons of the two delay maintenance tasks. In delay maintenance tasks, the small selection of corvids and psittacines tested—carrion crows, common ravens, Goffin's cockatoos, kea, African gray parrots—are able to inhibit consuming a low‐value reward to obtain a high‐value reward later, waiting for intervals comparable to primates (Figure 2). Some birds even do so while holding the initial reward in their mouths (Lambert et al., 2018). Corvids may select the smaller amount, as it is easier to eat, carry and cache nearby. However, as most primates have not been tested using quality based discriminations, it is difficult to interpret this further. The birds tend to tolerate long delays when waiting for a reward of higher quality; however, they struggle more in waiting for rewards of higher quantity (Auersperg et al., 2013; Hillemann et al., 2014). The birds also appeared to decide early in the trial whether to exchange (Dufour et al., 2012). Direct species comparisons have focussed primarily within taxa, for instance, indicating that great apes outperform other primate species (Dufour et al., 2007; Pelé et al., 2010; Pelé et al., 2011), rather than between primates and other groups, like birds.

**Table 2:** Performance in delay of gratification accumulation task indicated by maximum tolerated delay between items

Species		Maximum delay between items	Measure of success	Reference
Corvid	Common raven	320 s (quality: 640 s)	Maximum delay between items	(Hillemann et al., 2014)
	Carrion crow	40 s (quality: 40 s)	Maximum delay between items	(Hillemann et al., 2014)
Psittacine	African gray parrot	2 s	Number of accumulated items	(Vick et al., 2010)
Nonhuman primate	Chimpanzee	6 s	Number of accumulated items	(Evans et al., 2012)
	Chimpanzee	17 s	Maximum delay between items where they waited for 36 items	(Beran & Evans, 2006, 2006)
	Orangutan	6 s	Number of accumulated items, delay between first item and seizing items	(Parrish et al., 2014)
	Capuchin	9 s	Maximum delay between first item and seizing items	(Pelé et al., 2011)
	Capuchin	2 s	Number of accumulated items	(Evans et al., 2012)
	Capuchin	5 s	Number of accumulated items	(Anderson et al., 2010)
	Tonkean macaque	9 s	Maximum delay between first item and seizing items	(Pelé et al., 2011)
	Long‐tailed macaque	9 s	Maximum delay between first item and seizing items	(Pelé et al., 2010)
	Rhesus macaque	(quality: 120 s)	Maximum delay between items	(Evans & Beran, 2007)
	Squirrel monkey	5 s	Number of accumulated items	(Anderson et al., 2010)

*Note*. Quantity outcome reported unless specified otherwise. Note the differing measures of success across studies.

The limited number of corvid and psittacine species tested so far show similar socio‐ecology, for instance, most are group‐living, hence restricting evaluation of the influence of these factors on self‐control. Therefore, we would advocate testing other species within these taxa with differing socio‐ecological backgrounds, such as in sociality, caching, tool‐using behavior, would enable exploring their role. For example, comparing less social species, like Eurasian jay and Clark's nutcracker (*Nucifraga columbiana*), with highly social species, like rook.

## 3 CURRENT ISSUES AND FUTURE DIRECTIONS

In the following section, we discuss current issues in our understanding of delayed gratification as a measure of self‐control particularly across species that include nonprimates. We outline here, and in Box 2, future directions including how best to measure delayed gratification in order to comparatively investigate the drivers of this ability.

BOX 2 **OUTSTANDING QUESTIONS FOR FUTURE RESEARCH**

‐ Psychological mechanisms underpinning intertemporal choice in corvids and psittacines, as very little focus to date, despite extensive work in other taxa, particularly in humans.

‐ How do methodology and contextual alternations affect performance? For example, reward type, visibility and value, and experimenter familiarity.

‐ How do fission‐fusion dynamics influence self‐control rates? Like many primate species, corvids and psittacines have social systems with differing fission‐fusion dynamics, though no study has yet explored self‐control in bird groups with varying fission‐fusion levels.

‐ Few studies in nonhuman species testing the relation between self‐control, general intelligence, and the impact of self‐control abilities on measures of life success, despite extensive exploration in humans.

‐ Which cognitive and noncognitive factors influence self‐control, like motivation, body size, tool‐use, neophobia and caching? For example, more neophobic individuals/species may face a conflict between wanting to approach desired stimuli and fear preventing the approach in comparison with those less neophobic, influencing self‐control performance.

‐ How flexible is self‐control? Animals face variable environmental conditions that may influence whether it is most adaptive to select the immediate reward over a delayed reward, such as external pressures like predation risk, conspecific competition and perishable resources, or internal pressures, like hunger.

‐ How does social context influence self‐control? All studies reviewed involve self‐control in relation to inanimate stimuli—what about in a social situation, such as mate choice under competition, or reward choice when food‐sharing with a mate in food‐sharing species, like some corvids?

### 3.1 Methodological and contextual issues

In many cases, it is presently difficult to make fair species comparisons, as, even when using the same task paradigm, studies generally differ in various aspects, including sample sizes, number of trials, criterion for training and test trials, type of stimuli, reward type, quantity size differences, delay length/incremental increase and presentation of rewards. Additionally, there may be differences in: how subjects interact with the task, prior experience, motivation, reward value, amount of training and habituation, and level of involvement of the experimenter. Indeed, monkeys' performance in accumulation tasks varies across studies, potentially due to methodological issues (Addessi, Paglieri, & Focaroli, 2011; Paglieri et al., 2013). For instance, delay tolerance in capuchins increased considerably in delay choice compared to delay maintenance tasks (Addessi et al., 2013; Pelé et al., 2011). Furthermore, as reflected in the Supporting Information Tables, sample sizes are typically small, in some cases with only a few individuals tested, which introduces various issues relating to statistical power, reproducibility (Munafò et al., 2017) and generalizations to wider population/species. Similarly, with such small samples, it is unclear whether samples contain individuals across the possible spectrum of high to low self‐control.

At present, a major challenge in being able to assess delayed gratification abilities in different species in a comparative manner is considering how task performance can be evaluated comparatively. Accumulation studies typically report different outcome measures (Table 2), which limits direct species comparisons across groups, though does allow for describing the range of performances. Furthermore, in many delay maintenance studies, the outcome measure is maximum delay endured, which tends to only reflect performance of a few individuals waiting in a low number of trials, hence representing the behavior of single individuals rather than populations or species (Dufour et al., 2012; Hillemann et al., 2014). It is also unclear whether comparisons in maximum delay endured reflect biologically meaningful patterns. For example, in the qualitative exchange task, a carrion crow waited for 10 min, compared to an African gray parrot who waited for 15 min. The question arises whether the 5‐min difference reflects a biologically significant difference in delayed gratification ability, or reflects unrelated differences, such as methodology and prior subject experience. A similar point is now being recognized in the developmental literature, with reconsiderations of the marshmallow test, for example (Watts et al., 2018).

**Figure 3:**
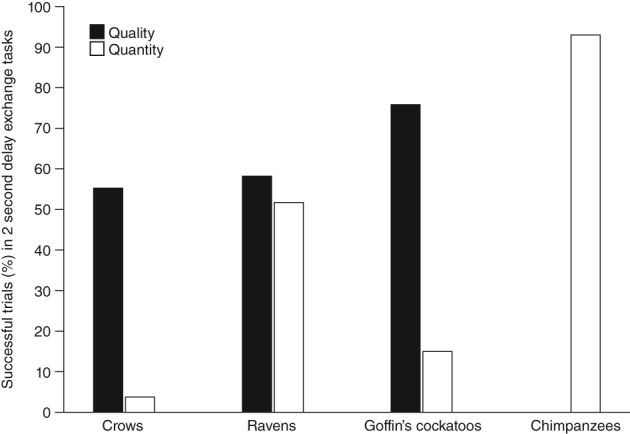
Percentage of successful trials in exchange tasks with a 2‐s delay when rewards differ in either quality (black) or quantity (white). Species selected reflects those studies where this data was published or made available from the original study authors (Auersperg et al., 2013; Dufour et al., 2007; Dufour et al., 2012; Hillemann et al., 2014)

Temporal discounting tasks address some of these concerns by generating equivalence points, which can be used to compare individuals, groups, species etc., (Green et al., 1997; Rosati et al., 2006), and are one approach to addressing some of the shortcomings of delay of gratification tasks. Additionally, we propose using an additional outcome measure of mean performance in early delay conditions, for example at 2 s. The advantage is that usually the entire subject group is tested in this condition, before they consecutively drop out of the experiment after they fail to wait in later conditions, thus giving a more representative measure of a species' rather than an individuals' ability to wait. Unfortunately, most studies at present only report maximum delay conditions reached, and not the focal subjects' mean performance at specific delay conditions. However, where available, we present the performance at 2‐s delay in exchange tasks (Figure 3; Table S4). Similar to performance measured by maximum delay (Figure 2), birds performed better in quality than quantity tasks. While the difference in performance was small for ravens, crows were almost 15‐times and cockatoos 5‐times more likely to exchange for a better‐quality than better‐quantity reward at a 2‐s delay. Chimpanzees, tested only using quantity discrimination, performed better than corvids and psittacines. We also found that, in crows and ravens (Dufour et al., 2012; Hillemann et al., 2014), individuals waiting in more trials in the 2‐s condition also sustained a higher maximum delay (Linear Mixed Effects Model: estimate ± SD: 0.082 ± 0.032, *t*‐value: 2.514, *p* = .001; Figure S1).

### 3.2 How best to measure delayed gratification?

Potential methodological issues should be taken into account in relation to the existing data, particularly when comparing species, but also in relation to future studies. However, before conducting further comparative research on delayed gratification as a measure of self‐control, we must consider how best to measure it. Performance across delayed gratification tasks correlates in adult humans, though not between delayed gratification and other executive function tasks (Duckworth & Kern, 2011). However, few studies have tested the same individuals/species in more than one delayed gratification task in nonhuman species, and some of those that have did not correlate performance across tasks. For example, in capuchins, there was only limited evidence of a significant correlation in intertemporal choice and accumulation performance (Addessi et al., 2013). In crows and ravens, we found no correlation between exchange and accumulation task performance (Linear Model: estimate ± SD: 1.332 ± 0.642, *t*‐value: 2.072, *p* = .071; Figure S2). Performance in different self‐control tasks may be mediated by different cognitive processes. However, given the few species and individuals tested on this at present, it is clear that the construct validity of different inhibitory control tasks requires further investigation, such as the neuro‐mechanisms influencing performance in different tasks (van Horik et al., 2018).

In order to address the question of construct validity, we need to identify existing tasks and develop novel tasks that show validity both (a) within and (b) between species. We suggest a step‐wise approach. The first step is to test delayed gratification tasks within‐subjects, ideally using procedures that can be standardized for different species, to identify the optimal tasks for testing self‐control by testing whether performance correlates across tasks on an individual and group level. If performance does correlate across tasks, this would indicate that delayed gratification as a measure of self‐control is a domain‐general cognitive ability with consistent individual differences across various contexts (Völter et al., 2018). Then these tasks would be likely to be worthwhile to explore in other species in a comparative manner, as they appear to be reliable self‐control measures. If performance does not correlate between tasks, then this would indicate that self‐control is not a unitary concept (Völter et al., 2018). Alternatively, it may be that some researchers use tasks that are erroneously referred to as self‐control tasks, when, in fact, noncorrelation between tasks may indicate that some tasks are not tests of self‐control. Voelter and colleagues recently suggested a similar approach in advocating the need to first establish convergent and divergent validity of cognitive tasks to assess whether they test one ability or several (Völter et al., 2018). They argue for a multitrait, multimethod approach with sufficient sample sizes, with a focus on consistency of individual performance.

As the second step, we advocate testing comparatively first across similar species (e.g., family/order) and then across species of different groups, like primates and birds. Both are important, as selecting only a small number of species to represent a whole taxonomic group is unlikely to be truly representative. This was highlighted recently in a large comparative study of motor‐self regulation, where the two corvid species tested (Eurasian jay, California scrub‐jay) performed relatively poorly in comparison to mammals (MacLean et al., 2014). However, when three other corvid species were tested (New Caledonian crows, ravens, jackdaws), they ranked top 10 with mammals (Kabadayi et al., 2017; Kabadayi et al., 2016).

This approach will clearly require some coordinated research effort to significantly boost sample sizes to increase power and species representations. Therefore, it is particularly important to save research time and effort by finding the optimal task within‐species, particularly as a task that is optimal for one species may not be optimal for all. Additionally, we would advocate the use of repeated‐measures tests to show reliability across assessments within the same individuals/species, for example obtaining the same equivalence points or maximum delay tolerance as a within‐subjects outcome (Beran, 2019, personal communication). The ability to delay gratification *should* be expected to show individual‐level consistency over time (Völter et al., 2018). Another benefit is that, once established, these self‐control measures would be available as a baseline measure for correlating with individual performance on other cognitive tasks (Box S1). For instance, motor‐self regulation performance was controlled for when comparing tool‐ and non‐tool using birds (Teschke et al., 2013). It would therefore be of benefit for future research in corvids, psittacines and other species to test for delayed gratification as a baseline measure for comparative cognition research.

### 3.3 Other delayed gratification tasks for future comparative research

In addition to the outstanding questions highlighted in Box 2, we provide an overview of several other delayed gratification paradigms that have received less focus than those discussed earlier, and may provide further avenues for future research. The hybrid delay task combines delay choice (choosing to wait) from delay maintenance (tolerating the delay) to test how often choosing to wait correlates with the ability to do so (Paglieri et al., 2013). A few primate species have been tested on this task. In capuchin monkeys (*n* = 18), subjects often chose to wait for the delayed reward, but failed to wait for it due to poor delay maintenance, though performance improved with experience (Paglieri et al., 2013). In chimpanzees (*n* = 40), performance in the hybrid delay task correlated with general intelligence (Beran & Hopkins, 2018). Findings of the hybrid delay task indicate that delay choice tasks may not assess self‐control, as choosing to wait may not correlate with the ability to do so (Paglieri et al., 2013). This task has not been tested in corvids or psittacines and only in few primate species, and therefore should be a focus for future research.

In the patch‐leaving task, subjects receive a smaller reward first and can then make the decision to either stay in a patch (i.e., a specific area within the testing arena) to wait for a second, larger reward, or leave the patch and immediately start the next trial (Table S5). It is essentially an accumulation task without intertrial intervals where animals are allowed to consume the first reward (Stephens & Anderson, 2001). Stephens and colleagues argue that delayed gratification tasks typically present a situation that does not occur in the animals' natural environment. Instead, they propose this patch‐leaving task, which has been used in a few corvid species, though not yet in other species. Though, following a similar idea, context influenced choice behavior in common marmosets and cotton‐top tamarins, when required to travel a further distance to obtain the larger reward (Stevens, Rosati, Ross, & Hauser, 2005).

Finally, as most delayed gratification tasks are time and effort intensive, for example, extensive pretraining and close interaction with an experimenter is typically required for token‐exchange tasks (Dufour et al., 2012), in addition to the more traditional paradigms, we advocate designing novel automated paradigms. For example, it would be of interest to explore delayed gratification with quality and quantity‐based discriminations using the automated rotating tray task, which requires minimal pretest training (Bramlett et al., 2012). This would negate the need for more complex pretraining. Another example would be automated feeder tasks, such as a touch‐screen for delay choice tasks, or a reward‐dispenser in accumulation tasks. Similarly, it would negate any potential concerns regarding a bias towards species/individuals with extensive experimental experience or higher experimenter familiarity over those without. Individuals of the same species could then be tested using both automated and experimenter interaction set‐ups to explore whether there are any performance differences. For instance, comparing performance using the automated rotating tray with that of more traditional methodologies, in a similar manner to a previous capuchin study (Beran, Perdue, et al., 2016).

### 3.4 Evolutionary explanations for delayed gratification

“Good” self‐control in humans and other animals is generally thought to be beneficial, for instance, delayed gratification in children correlates with behavioral outcomes in later life (Mischel et al., 1989). However, there is also research that suggests that temporal impulsivity may be adaptive under certain circumstances (Fawcett et al., 2012; Stephens, Kerr, & Fernández‐Juricic, 2004), such as in times of high competition over resources. For example, delayed gratification performance is influenced by environmental stability in children (Kidd et al., 2013). As we highlight in Box 2, further exploration of the flexibility of delayed gratification under differing social and physical conditions in corvids and psittacines—and other taxa—would be beneficial. Social paradigms indicate that in humans, the presence and behavior of others can influence decision‐making. Flexibility in self‐control is likely to be important in a social context in nonhuman animals too, for instance, refraining from approaching food or a potential mate while in the presence of a competitor (Bugnyar, 2011; Duque & Stevens, 2017). Despite this, there are only a few tasks that require interaction with a conspecific, which are primarily the exchange paradigms in primates (Table S1). Therefore, there is scope for developing social tasks that also explore the flexibility of self‐control, for instance by focussing on the effect of dominance on delayed gratification performance. Indeed, high‐ranking capuchin monkeys, though not low‐ranking ones, quickly acquired token‐exchange behavior in social contexts (Addessi et al., 2011).

### 3.5 What are the drivers of delayed gratification?

Once the construct validity of delayed gratification as a measure of self‐control is established, this measure can be used to assess the potential drivers for self‐control. Here, we argue that corvids, psittacines and nonhuman primates provide a unique opportunity to investigate specific socio‐ecological factors shaping the evolution for self‐control in these disparate groups. We propose different strategies for investigating whether ecological aspects (like caching, tool‐use) and social characteristics (like fission‐fusion) are key factors in the evolution of self‐control. Caching behavior may improve self‐control, as caching species must inhibit eating food items immediately. It would therefore be useful to compare caching species (e.g., ravens, crows, and jays) and species that do not cache or do so less routinely (e.g., jackdaws, rooks), which has not yet been done. Furthermore, tool‐use is likely to require some degree of inhibitory control. Indeed, tool‐using capuchins outperform nontool using common marmosets and cotton‐top tamarins (Addessi et al., 2011). Tool‐using New Caledonian crows do not outperform nontool using carrion crows in a motor‐self regulation task (Teschke et al., 2013). Tests comparing delayed gratification ability between tool‐using species (e.g., chimpanzees, capuchins, New Caledonian crows, Hawaiian crows *Corvus hawaiiensis*) versus nontool using species (e.g., marmosets, gray parrots, common ravens, carrion crows) across these three groups is desirable.

In nonhuman primates, species differences in self‐control have been attributed to varying fission‐fusion dynamics (Amici et al., 2008). In corvids, sociality correlates with a motor self‐regulation task performance in three corvid species (Bond et al., 2007). However, the role of social system, such as fission‐fusion dynamics, on self‐control has yet to be explored in corvids and psittacines. Additionally, other factors should be controlled for when comparing different groups. For example, in primates, larger species tolerating longer delays than smaller species, potentially because larger species have lower metabolism and longer life expectancies (Stevens & Mühlhoff, 2012).

Through this review, we wish to highlight that specific socio‐ecological factors may correlate with increased delayed gratification abilities. In general, high degrees of delayed gratification may have evolved in contexts where the species relied on variable food resources that are difficult to retrieve, or risky to obtain in feeding situations, leading to competition and cooperation for food resources. Alongside cognitively complex abilities in this species, these pressures may drive extractive foraging, including the capacity for tool‐use, or scattered, large food bonanzas leading to caching. While it is unclear whether self‐control aids in these behaviors, these pressures lead to enhanced delayed gratification, it is important to improve our understanding of these relations.

## 4 CONCLUSION

Corvids and psittacines show a capacity for delayed gratification that is comparable to that of primates. As discussed, however, further studies are required to allow for crucial comparisons to be made that take into account methodological constraints. We suggest potential avenues for future research, which include how best to measure delayed gratification and explore species differences in delayed gratification comparatively across different taxa, in order to better identify the evolutionary drivers of self‐control and the way in which they may be enhanced and/or constrained by socio‐ecological factors.

Self‐control is critical, aiding in other cognitive processes including decision making, planning and goal‐oriented behavior (also see Box S1 on cognition and inhibitory control). For example, it is thought to be an important cognitive prerequisite of cooperation, as individuals may help a conspecific now to be able to receive a favor in future (Stevens & Hauser, 2004). We therefore need to understand self‐control in order to fully comprehend cognition, particularly when comparing across species from different taxa. Our review provides an essential research framework, highlighting the need to identify the best means of testing self‐control and compiling broad data sets systematically that in turn enable comprehensive meta‐analyses, which will allow us to address open questions regarding the evolution of cognition.

## CONFLICT OF INTEREST

The authors have declared no conflicts of interest for this article.

## RELATED WIREs ARTICLE

https://doi.org/10.1002/wcs.1504

## Supporting information


**Data S1.** Supporting Information
