# Supplementary material for: Self‐control in crows, parrots and nonhuman primates
Source: Wiley Interdiscip Rev Cogn Sci. 2019 May 20;10(6):e1504. doi: 10.1002/wcs.1504 (PMC6852083; doi:10.1002/wcs.1504)
Supplement: Supplementary file 1 — Data S1. Supporting Information [file WCS-10-na-s001.docx]

**Supplementary Information**

**Self-control in crows, parrots and non-human primates**

Rachael Miller^1*^, Markus Boeckle^1,2,3^, Sarah A. Jelbert^1^, Anna Frohnwieser^1^, Claudia A.F. Wascher^4^, Nicola S. Clayton^1^

^1^ Department of Psychology, University of Cambridge, United Kingdom

^2^ Department of Cognitive Biology, University of Vienna, Austria

^3^ Department of Psychotherapy, Bertha von Suttner University, Austria

^4^ School of Life Sciences, Anglia Ruskin University, United Kingdom

Box S1. Cognition and inhibitory control

| Most previous comparative cognitive research in non-human animals aims to understand complex cognition without considering inhibitory control abilities (e.g.: Maclean et al., 2014), despite many cognitive tasks relying on inhibition of reflex-like or conditioned behaviours before more complex behavioural patterns can be produced. Differences in performance between species, may reflect differences in cognitive abilities, or may relate to inhibition issues leading to a failure to inhibit selection of stimuli previously associated with food (Miller et al., 2016). For example, great apes perform poorly in comparison to children in false belief tasks, which aim to test the ability to recognise that others can have different beliefs than one’s own (Call & Tomasello, 1999). Specifically, 5-year old children are able to correctly locate a reward hidden in one of two containers by a ‘hider’ adult, by reasoning that a second adult not present when the hider moved the reward to the other container had not observed it being moved, while younger children and great apes failed (Call & Tomasello, 1999). In another example, following training to prefer a non-functional object, New Caledonian crows (*Corvus moneduloides*) though not 5 to 7-year old children, performed significantly more poorly in subsequent object-choice water displacement tasks, than subjects in previous studies (Miller et al., 2016). This may be particularly relevant for cognitive tasks that require making choices between two stimuli, where one option is associated with a reward. This distinction is especially important when investigating tasks comparing cognitive aspects across species, particularly given recent findings that, like in children (Mischel, Shoda, & Rodriguez, 1989), self-control relates to general intelligence in chimpanzees (*Pan troglodytes*). These findings highlight the key role of inhibitory, cognitive processes in general intelligence (Beran & Hopkins, 2018). Hence, species and individuals with better self-control skills may appear to outperform those with poorer skills as they may have higher flexibility in their responses to cognitive tasks, rather than differing in cognitive abilities. |
| --- |

Box S2. The role of distraction in delay of gratification studies

| Distraction is a strategy that can improve self-control. Chimpanzees often utilise distraction techniques during the delay. For instance, playing with toys when the accumulation tube was available would increase their delay tolerance in comparison to when no toys were present or if toys were present but the tube was not (Evans & Beran, 2007). Children that distracted themselves during the delay, such as touching or playing with the cookie, talking to the parent or playing, tended to exchange more successfully, compared to those with a passive waiting attitude (Steelandt, Thierry, Broihanne, & Dufour, 2012). Carrion crows (*Corvus corone*) and common ravens (*Corvus corax*) employ tactics, such as placing the reward on the ground or caching it, in order to alleviate the costs of waiting and distract their attention during delays (Dufour, Wascher, Braun, Miller, & Bugnyar, 2012). Keas (*Nestor notabilis*) were able to achieve longer wait times for better quality food rewards by being provided with a container that the first reward could be kept in during the delay, instead of having to keep it in their beaks (Schwing, Weber, & Bugnyar, 2017). |
| --- |

Table S1. Self-control performance in social context using delay of gratification exchange paradigm

| **Species** | **No. of subjects** | **Outcome (e.g. tested species differences)** | **References** |
| --- | --- | --- | --- |
| Common raven | 8 | Aiming to explore pro-sociality (exchanging individual did not receive reward for transferring token to conspecific), in conditions with conspecific or group, subjects did not regularly help conspecifics to gain access to food, though some spontaneous helping acts did occur. | (Massen, Lambert, Schiestl, & Bugnyar, 2015) |
| Chimpanzee | 4 | Learned to exchange tokens with a conspecific (or a computer) in order to accumulate rewards. Chose to engage in exchange task over free food. | (Parrish, Perdue, Evans, & Beran, 2013) |
| Chimpanzee; Gorilla (*Gorilla gorilla*)*;*  Bonobo *(Pan paniscus)*; Orangutans (*Pongo pygmaeus*);  Capuchin monkey; Tonkean macaque (*Macaca tonkeana*) | 4;  3;  5;  5;  9;  10 | Transferred tokens with conspecifics, mostly indirectly. Orangutans actively exchanged tokens with conspecific, whereas capuchin and Tonkean macaques made passive exchanges | (Addessi, Paglieri, & Focaroli; Pelé, Dufour, Thierry, & Call, 2009; Pelé, Micheletta, Uhlrich, Thierry, & Dufour, 2011) |
| Capuchin monkey | 8 | Transferred tokens and tools with conspecific, selected situation appropriate tokens to exchange for specific tools, though did not select appropriate tools in response to partner’s transferred token | (Westergaard, Evans, & Howell, 2007; Westergaard, Liv, Rocca, Cleveland, & Suomi, 2004) |
| Chimpanzee;  Bonobo;  Orangutan | 6;  5;  4 | In inequity aversion versions of task, subject received less-preferred reward in exchange for token, while competitor conspecific received same type or more preferred reward. Subjects did not refuse less-preferred food when competitor received more-preferred one | (Bräuer, Call, & Tomasello, 2009) |
| Capuchin monkey | 4 | In inequity aversion versions of task, no evidence of inequity aversion with respect to work effort of conspecific or inequity of food reward. Did not differentiate between ‘equal’ or ‘unequal’ experimenters | (Fontenot, Watson, Roberts, & Miller, 2007) (Sheskin, Ashayeri, Skerry, & Santos, 2014) |

Table S2. Performance in delay of gratification exchange task using maximum delay as the outcome measure. - = not tested. Note that, unlike other studies, in the African grey parrot study, the subject was verbally instructed to wait.

| **Species** | | **No. of subjects** | **Quality** | **Quantity** | **Reference** |
| --- | --- | --- | --- | --- | --- |
| Corvid | Carrion crow | 10 | 640s | 320s | (Dufour et al., 2012; Hillemann, Bugnyar, Kotrschal, & Wascher, 2014) |
|  | Common raven | 4 | 160s | 10s | (Dufour et al., 2012; Hillemann et al., 2014) |
| Psittacine | Goffin cockatoo (*Cacatua goffiniana*) | 14 | 80s | 20s | (Auersperg, Laumer, & Bugnyar, 2013) |
|  | Kea | 10 | 160s | 20s | (Schwing et al., 2017) |
|  | African grey parrot (*Psittacus erithacus*) | 1 | 15min | - | (Koepke, Gray, & Pepperberg, 2015) |
| Non-human primate | Chimpanzee | 5 | - | 4min; 8min for large quantity difference | (Dufour, Pelé, Sterck, & Thierry, 2007) |
|  | Capuchin monkey;  Tonkean macaque | 9;  10 | - | Capuchins: 40s; 21min when reward could be returned; Macaques: 80s; 43min when reward could be returned | (Pelé et al., 2011) |
|  | Long-tailed macaque (*Macaca fascicularis*) | 9 | - | 10min | (Pelé, Dufour, Micheletta, & Thierry, 2010) |

Table S3. Intertemporal choice task outcomes

| **Species** | | **No. of subjects** | **Outcome** | **Reference** |
| --- | --- | --- | --- | --- |
| Corvid | Californian scrub-jay (*Aphelocoma californica*) | 8 | Caching paradigm with delays up to 49 hours. No evidence of a preference for earlier recovery | (Thom & Clayton, 2014) |
| Non-human primate | Chimpanzee | 3 | Wait several minutes for reward of better quality | (Beran, Savage‐Rumbaugh, Pate, & Rumbaugh, 1999) |
|  | Chimpanzee;  Orangutan | 2;  1 | Select a tool (hose) from a choice of fruit and several toys to use 70 minutes later to consume a fruit soup | (Osvath & Osvath, 2008) |
|  | Chimpanzee;  Bonobo;  Capuchin monkey; Common marmoset (*Callithrix jacchus*);  Cotton-top tamarin (*Saguinus oedipus*);  Black and white ruffed lemur (*Varecia variegata*);  Red-ruffed lemur (*Varecia rubra*);  Black lemur (*Eulemur macaco*) | 5-19;  5;  18;  5;  6;  4;  2;  2 | Capuchins higher tolerance with reward of higher quantity than marmosets and tamarins, and comparable to chimpanzees and bonobos, may be related to tool-using abilities in capuchins. Marmosets waited longer than tamarins for a larger reward Lemurs were comparable to marmosets, with capuchins waiting three times longer than lemurs | (Stevens, Hallinan, & Hauser, 2005) (Rosati, Stevens, Hare, & Hauser, 2007; Stevens, 2014; Stevens & Mühlhoff, 2012) |
|  | Capuchin monkey | 8 | Successful when reward of higher quality and quantity is presented on rotating tray, learn to let first reward go past to wait for second one that is bigger/ better | (Bramlett, Perdue, Evans, & Beran, 2012) |

Table S4. Performance in exchange tasks with a 2-second delay when rewards differ in either quality or quantity. We have included species from studies where this data was available, or where it was provided by the original study authors. Note that the quantity difference is typically combined in the results sectio of these studies, as most studies found no differences in performance depending on the quantity difference. - = not applicable

| **Species** | **No. of subjects** | **Success (%) in 2-second delay** | | **Reference** |
| --- | --- | --- | --- | --- |
|  |  | Quality exchange | Quantity exchange |  |
| Carrion crow | 10 | 55.12 | 3.74 | (Dufour et al., 2012; Hillemann et al., 2014) |
| Common raven | 4 | 58.03 | 51.57 | (Dufour et al., 2012; Hillemann et al., 2014) |
| Goffin cockatoo | 14 | 75.71 | 14.88 | (Auersperg et al., 2013) |
| Chimpanzee | 5 | - | 93 | (Dufour et al., 2007) |
| Dog (*Canis familiaris*) | 5 | - | 78 | (Leonardi, Vick, & Dufour, 2012) |

Table S5. Patch-leaving task, tested only in corvids to date.

| **Species** | **No. of subjects** | **Outcome** | **References** |
| --- | --- | --- | --- |
| Blue jay (*Cyanocitta cristata*) | 8 | Perform better in this task then other self-control task in terms of optimising long-term food intake | (Stephens & Dunlap, 2009) |
| Pinyon jay (*Gymnorhinus cyanocephalus*) | 12 | Wait mean delay of 8 seconds for quantity, no relationship between performance in task and how much food cached | (Stevens, Kennedy, Morales, & Burks, 2016) |
| Californian scrub-jay (*Aphelocoma californica*) | 8-14 | No preference to cache in locations that can be revisited sooner than later, though adjusted food types cached depending on whether they degraded before recovery or not | (Clayton, Dally, Gilbert, & Dickinson, 2005; Thom & Clayton, 2014) |


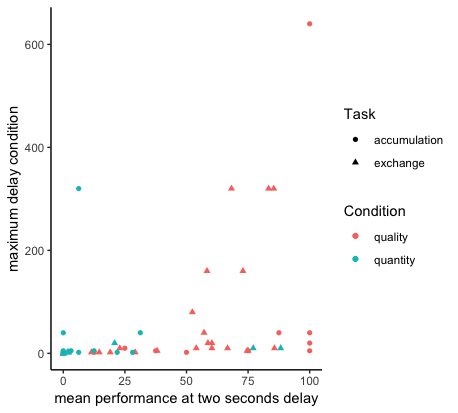


Figure S1: Mean performance of corvids in the two second delay condition (x-axis) compared to maximum delay condition reached (y-axis) in the accumulation and exchange task. Data from (Dufour et al., 2012; Hillemann et al., 2014).

**
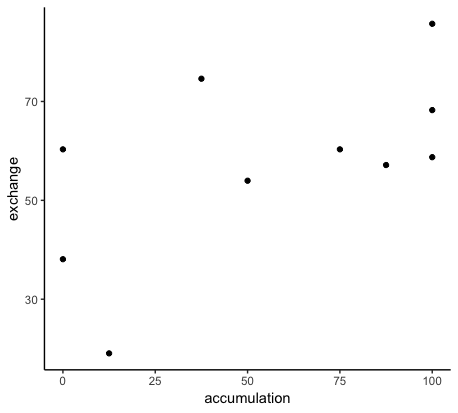
**

Figure S2: Mean performance of corvids in the two second delay condition in the qualitative accumulation (x-axis) compared to qualitative exchange task (y-axis). Data from (Dufour et al., 2012; Hillemann et al., 2014).

**Supplementary References**

Addessi, E., Paglieri, F., & Focaroli, V. (2011). The ecological rationality of delay tolerance: insights from capuchin monkeys. *Cognition, 119*(1), 142-147.

Auersperg, A. M., Laumer, I., & Bugnyar, T. (2013). Goffin cockatoos wait for qualitative and quantitative gains but prefer ‘better’to ‘more’. *Biology Letters, 9*(3), 20121092.

Beran, M. J., & Hopkins, W. D. (2018). Self-control in chimpanzees relates to general intelligence. *Current Biology, 28*(4), 574-579. e573.

Beran, M. J., Savage‐Rumbaugh, E. S., Pate, J. L., & Rumbaugh, D. M. (1999). Delay of gratification in chimpanzees (Pan troglodytes). *Developmental Psychobiology: The Journal of the International Society for Developmental Psychobiology, 34*(2), 119-127.

Bramlett, J. L., Perdue, B. M., Evans, T. A., & Beran, M. J. (2012). Capuchin monkeys (Cebus apella) let lesser rewards pass them by to get better rewards. *Animal cognition, 15*(5), 963-969.

Bräuer, J., Call, J., & Tomasello, M. (2009). Are apes inequity averse? New data on the token‐exchange paradigm. *American Journal of Primatology, 71*(2), 175-181.

Call, J., & Tomasello, M. (1999). A nonverbal false belief task: The performance of children and great apes. *Child development, 70*(2), 381-395.

Clayton, N. S., Dally, J., Gilbert, J., & Dickinson, A. (2005). Food caching by western scrub-jays (Aphelocoma californica) is sensitive to the conditions at recovery. *Journal of Experimental Psychology: Animal Behavior Processes, 31*(2), 115.

Dufour, V., Pelé, M., Sterck, E., & Thierry, B. (2007). Chimpanzee (Pan troglodytes) anticipation of food return: coping with waiting time in an exchange task. *Journal of Comparative Psychology, 121*(2), 145.

Dufour, V., Wascher, C. A., Braun, A., Miller, R., & Bugnyar, T. (2012). Corvids can decide if a future exchange is worth waiting for. *Biology Letters*, rsbl20110726.

Evans, T. A., & Beran, M. J. (2007). Chimpanzees use self-distraction to cope with impulsivity. *Biology Letters, 3*(6), 599-602.

Fontenot, M., Watson, S., Roberts, K., & Miller, R. (2007). Effects of food preferences on token exchange and behavioural responses to inequality in tufted capuchin monkeys, Cebus apella. *Animal Behaviour, 74*(3), 487-496.

Hillemann, F., Bugnyar, T., Kotrschal, K., & Wascher, C. A. (2014). Waiting for better, not for more: corvids respond to quality in two delay maintenance tasks. *Animal Behaviour, 90*, 1-10.

Koepke, A. E., Gray, S. L., & Pepperberg, I. M. (2015). Delayed gratification: A grey parrot (Psittacus erithacus) will wait for a better reward. *Journal of Comparative Psychology, 129*(4), 339.

Leonardi, R. J., Vick, S.-J., & Dufour, V. (2012). Waiting for more: the performance of domestic dogs (Canis familiaris) on exchange tasks. *Animal cognition, 15*(1), 107-120.

Maclean, E. L., Hare, B., Nunn, C. L., Addessi, E., Amici, F., Anderson, R. C., . . . Zhao, Y. (2014). The evolution of self-control. *Proc Natl Acad Sci U S A*. Retrieved from <http://www.ncbi.nlm.nih.gov/pubmed/24753565>. doi:10.1073/pnas.1323533111

Massen, J. J., Lambert, M., Schiestl, M., & Bugnyar, T. (2015). Subadult ravens generally don't transfer valuable tokens to conspecifics when there is nothing to gain for themselves. *Frontiers in psychology, 6*, 885.

Miller, R., Jelbert, S. A., Taylor, A. H., Cheke, L. G., Gray, R. D., Loissel, E., & Clayton, N. S. (2016). Performance in Object-Choice Aesop's Fable Tasks Are Influenced by Object Biases in New Caledonian Crows but not in Human Children. *Plos one, 11*(12). Retrieved from <Go to ISI>://WOS:000389587100265. doi:ARTN e0168056

10.1371/journal.pone.0168056

Mischel, W., Shoda, Y., & Rodriguez, M. I. (1989). Delay of gratification in children. *Science, 244*(4907), 933-938.

Osvath, M., & Osvath, H. (2008). Chimpanzee (Pan troglodytes) and orangutan (Pongo abelii) forethought: self-control and pre-experience in the face of future tool use. *Animal cognition, 11*(4), 661-674.

Parrish, A. E., Perdue, B. M., Evans, T. A., & Beran, M. J. (2013). Chimpanzees (Pan troglodytes) transfer tokens repeatedly with a partner to accumulate rewards in a self-control task. *Animal cognition, 16*(4), 627-636.

Pelé, M., Dufour, V., Micheletta, J., & Thierry, B. (2010). Long-tailed macaques display unexpected waiting abilities in exchange tasks. *Animal cognition, 13*(2), 263-271.

Pelé, M., Dufour, V., Thierry, B., & Call, J. (2009). Token transfers among great apes (Gorilla gorilla, Pongo pygmaeus, Pan paniscus, and Pan troglodytes): species differences, gestural requests, and reciprocal exchange. *Journal of Comparative Psychology, 123*(4), 375.

Pelé, M., Micheletta, J., Uhlrich, P., Thierry, B., & Dufour, V. (2011). Delay maintenance in Tonkean macaques (Macaca tonkeana) and brown capuchin monkeys (Cebus apella). *International Journal of Primatology, 32*(1), 149-166.

Rosati, A. G., Stevens, J. R., Hare, B., & Hauser, M. D. (2007). The evolutionary origins of human patience: Temporal preferences in chimpanzees, bonobos, and human adults. *Current Biology, 17*(19), 1663-1668. Retrieved from <Go to ISI>://WOS:000250125200025. doi:10.1016/j.cub.2007.08.033

Schwing, R., Weber, S., & Bugnyar, T. (2017). Kea (Nestor notabilis) decide early when to wait in food exchange task. *Journal of Comparative Psychology, 131*(4), 269.

Sheskin, M., Ashayeri, K., Skerry, A., & Santos, L. R. (2014). Capuchin monkeys (Cebus apella) fail to show inequality aversion in a no-cost situation. *Evolution and Human Behavior, 35*(2), 80-88.

Steelandt, S., Thierry, B., Broihanne, M.-H., & Dufour, V. (2012). The ability of children to delay gratification in an exchange task. *Cognition, 122*(3), 416-425.

Stephens, D. W., & Dunlap, A. S. (2009). Why do animals make better choices in patch-leaving problems? *Behavioural processes, 80*(3), 252-260.

Stevens, J. R. (2014). Evolutionary pressures on primate intertemporal choice. *Proceedings of the Royal Society of London B: Biological Sciences, 281*(1786), 20140499.

Stevens, J. R., Hallinan, E. V., & Hauser, M. D. (2005). The ecology and evolution of patience in two New World monkeys. *Biology Letters, 1*(2), 223-226.

Stevens, J. R., Kennedy, B. A., Morales, D., & Burks, M. (2016). The domain specificity of intertemporal choice in pinyon jays. *Psychonomic bulletin & review, 23*(3), 915-921.

Stevens, J. R., & Mühlhoff, N. (2012). Intertemporal choice in lemurs. *Behavioural processes, 89*(2), 121-127.

Thom, J. M., & Clayton, N. S. (2014). No evidence of temporal preferences in caching by Western scrub-jays (Aphelocoma californica). *Behavioural processes, 103*, 173-179.

Westergaard, G. C., Evans, T. A., & Howell, S. (2007). Token mediated tool exchange between tufted capuchin monkeys (Cebus apella). *Animal cognition, 10*(4), 407-414.

Westergaard, G. C., Liv, C., Rocca, A. M., Cleveland, A., & Suomi, S. J. (2004). Tufted capuchins (Cebus apella) attribute value to foods and tools during voluntary exchanges with humans. *Animal cognition, 7*(1), 19-24.
